# Diet-Induced Obesity Mice Execute Pulmonary Cell Apoptosis via Death Receptor and ER-Stress Pathways after *E. coli* Infection

**DOI:** 10.1155/2020/6829271

**Published:** 2020-06-28

**Authors:** Fengyuan Wang, Zhicai Zuo, Kejie Chen, Jing Fang, Hengmin Cui, Yi Geng, Ping Ouyang, Zhengli Chen, Chao Huang, Hongrui Guo, Wentao Liu

**Affiliations:** ^1^College of Veterinary Medicine, Sichuan Agricultural University, Chengdu, Sichuan 611130, China; ^2^College of Life Science and Technology, Southwest Minzu University, Chengdu, Sichuan 610041, China; ^3^School of Public Health, Chengdu Medical College, Chengdu, Sichuan 610500, China

## Abstract

Obesity has developed into a considerable health problem in the whole world. *Escherichia coli* (*E. coli*) can cause nosocomial pneumonia and induce cell apoptosis during injury and infection. Normal (lean) and diet-induced obesity mice (DIO, fed with high-fat diet) were chosen to perform nasal instillation with *E. coli* to establish a nonfatal acute pneumonia model. At 0 h, 12 h, 24 h, and 72 h postinfection, lung tissues were obtained to measure cell apoptosis. As shown in this study, both lean and DIO mice exhibited histopathological lesions of acute pneumonia and increased cell apoptosis in the lung infected with *E. coli*. Interestingly, the relative mRNA and protein expressions associated with either endoplasmic reticulum stress or death receptor apoptotic pathway were all dramatically increased in the DIO mice after infection, while only significant upregulation of death receptor apoptotic pathway in the lean mice at 72 h. These results indicated that the DIO mice executed excess cell apoptosis in the nonfatal acute pneumonia induced by *E. coli* infection through endoplasmic reticulum stress and death receptor apoptotic pathway.

## 1. Introduction

Pneumonia has been recognized as a common cause of sepsis in critically ill patients today [1]. More than 60% of nosocomial pneumonias are caused by gram-negative enteric bacilli [2]. *Escherichia coli* (*E. coli*), a rod-shaped gram-negative bacterium, could produce disease of organ systems other than the gut, including urinary tract infection, meningitis, septicemia, severe community-, and ventilator-acquired pneumonia in humans and animals [3–6]. To combat pathogen, innate immune responses initiate the release of proinflammatory cytokines and recruitment of inflammatory cells [7]. Neutrophils are the first circulating leukocytes to respond during *E. coli* pneumonia [8], and its apoptosis after killing pathogen is considered to be essential for the downregulation of inflammatory response [9].

Apoptosis is an essential event in normal life and development. When stimulating factors persist, apoptotic signaling pathways are initiated and damaged cells are eliminated [10]. The TNF- (tumor necrosis factor-) induced model and Fas-Fas ligand-mediated model are extrinsic signals both involving receptors of the TNF receptor (TNFR) family [11]. TNF-*α*, a major extrinsic mediator of apoptosis, is a cytokine produced mainly by activated macrophages. TNF-related apoptosis plays an important role in the orchestration of the innate immune responses [12]. In conditions of prolonged stress, oxidative stress may have an interaction with endoplasmic reticulum stress, and influence unfolded protein response (UPR) signaling activity that commits the cell to a pathway of apoptosis [13]. As reported previously, virus-induced acute lung injury could activate the endoplasmic reticulum (ER) stress-induced apoptotic pathway, such as upregulated expressions of PERK, CHOP, *p-*JNK, and Caspase-12 [14], and *Mycobacterium bovis* has been reported to induce macrophages apoptosis through ER stress-mediated pathway by activating IRF3 [15].

In general, for adults, the lungs of obese individuals exhibit impaired function, including reduced lung volume and expiratory flow rates [16], which might be more vulnerable to bacterial invasion. However, according to our previous study, the DIO mice exhibited a delayed inflammatory response and oxidative stress, as well as pulmonary cell apoptosis through the mitochondria-mediated pathway [17]. In order to have a more integrated understanding of the differences in pulmonary cell apoptosis between normal and obese mice during acute bacterial pneumonia, by the diet-induced obesity and acute pneumonia model, we detected the effect of diet-induced obesity on ER stress- and death receptor-mediated apoptosis in the setting of acute pneumonia.

## 2. Materials and Methods

### 2.1. Animal Model of Obesity and Acute Pulmonary Infection

128 male ICR mice, after fed with normal or high-fat diet for 8 weeks, were divided into 4 groups (32/group), named lean-*E. coli*, lean-uninfected, DIO-*E. coli*, and DIO-uninfected followed by instilled intranasally with *Escherichia coli* (*E. coli*) or PBS using the published protocol [17, 18]. At 0 h, 12 h, 24 h, and 72 h, eight mice from each group were euthanized, and the left lungs were preserved in 4% paraformaldehyde, while the right ones were kept in liquid nitrogen. The compositions of the experimental diets were in accordance with a previous study [19]. All experimental procedures were performed in accordance with the national and international guidelines and regulations and were approved by the Sichuan Agricultural University Animal Care and Use Committee (Approval No: 2012-024).

### 2.2. Lung Injury Assayed by Histopathology

At 12 h after infection, tissue slices of left lungs were made traditionally and processed hematoxylin and eosin staining. Histopathological changes were observed and photographed with a digital camera (Nikon DS-Ri1, Japan).

### 2.3. TUNEL Assay

At indicated time points of the experiment, the dewaxing lung paraffin sections underwent the TUNEL assay according to the manufacturer's instruction of the Apoptosis Detection Kit (Boster Corporation, China, MK1020). The numbers of TUNEL-positive cells were calculated at 400× magnification of 5 fields/image with a digital microscope camera system (Nikon DS-Ri1, Japan) and analyzed using computer-assisted image-Pro Plus 5.1 (USA) as previously described [20].

### 2.4. Quantitative Real-Time PCR

Total RNA of frozen right lungs from eight mice in each group were prepared from Trizol extracts. Then, they were reverse transcribed and amplified with primers specific for target factors and *β*-actin (Table 1) using methods similar to previous documents. Gene expression values from the control group subsamples at 0 h were used to calibrate gene expression in subsamples from corresponding experimental subsamples. All data outputs from the qRT-PCR experiments were analyzed using the 2^-*ΔΔ*CT^ method. The primers were synthesized at Sangon biotech (Shanghai, China).

### 2.5. Western Blotting

The proteins of the right lungs were extracted with RIPA lysis buffer and separated by SDS-PAGE (15% gels). After electrophoresis, the proteins were transferred onto nitrocellulose membranes followed by 5% nonfat dry milk blocking and the primary antibodies incubating overnight at 4°C. The primary antibodies were Calpain 2, TNFR1, FADD, Caspase 12, JNK, TNF-*α*, and GAPDH (Abcam, ab126600, ab109322, and Cell signaling technology, 2202, 9252, 11948, and 5174). The blots were developed with the biotin-conjugated secondary antibodies (Cell signaling technology, 7074) and visualized by ECL™ (Beyotime technology, P0018A). Then, the statistical data of protein expressions were done with Quantity One software.

### 2.6. Immunohistochemistry

The dewaxing lung paraffin sections acquired were treated by 3.0% hydrogen peroxide blocking, boiling sodium citrate solution retrieval, 5% BSA blocking, and primary antibody (anti-Calpain 2, anti-Caspase 12, anti-JNK, anti-TNF-*α*, anti-TNFR1, or anti-FADD incubating overnight at 4°C, respectively). Then, the sections were developed with SABC kits using protocols provided by the manufacturer (Wuhan Boster Bio-engineering Limited Company, China, SA1020). The positive proteins were finally visualized by DAB and photographed with a digital camera (Nikon DS-Ri1, Japan).

### 2.7. Statistical Analysis

The SPSS 17.0 statistical software package programme for Windows was used for statistical tests. All results were expressed as Mean ± Standard Deviation. Differences between group means were estimated using LSD or Dunnett's T3 by one-way analysis of variance (ANOVA). A value of *p* < 0.05 was considered as statistically significant differences. The change rate was calculated by the following formula, and DIO and lean in the figures indicated the change rate of DIO and lean mice, respectively.

(1) $$\begin{array}{c} \text{Change rate }\left(\%\right)=\frac{\text{value of infected mice}-\text{value of uninfected mice }}{\text{value of uninfected mice}}\times 100\%. \end{array}$$

## 3. Results

### 3.1. Pathological Injuries of Lungs

**Figure 1:**
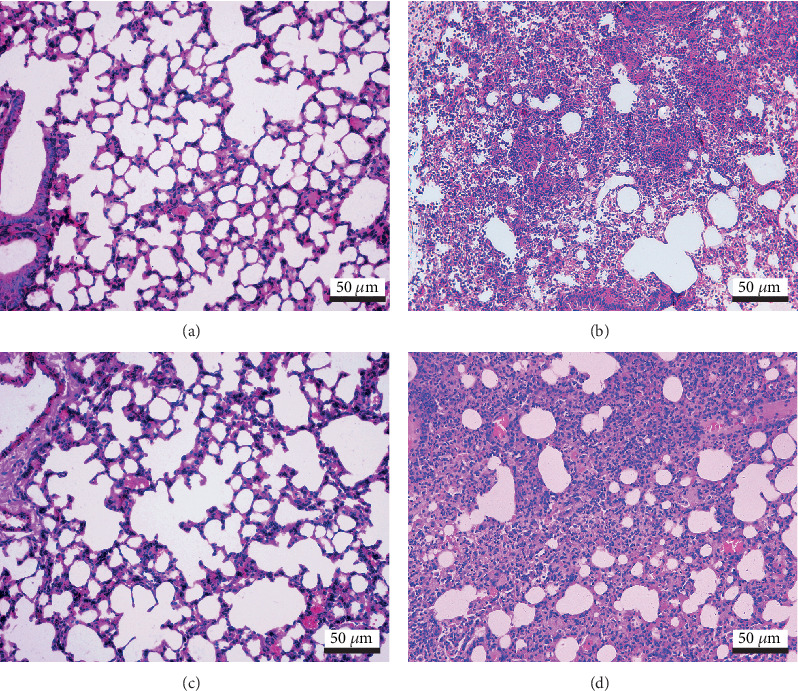
The representative histopathological changes of lung at 12 h postinfection. (a) Lean-uninfected group; (b) lean-*E. coli* group; (c) DIO-uninfected group; (d) DIO-*E. coli* group. H.E. Stain, scale bar = 50 *μ*m.

As shown in Figure 1, a huge number of inflammatory cells (including neutrophils, lymphocytes, and macrophages) infiltrated into the bronchioles and alveolus, alveolar wall, arose hyperaemia, and hemorrhage, and caused an acute inflammation in both the lean- and DIO-*E. coli* groups at 12 h after infection.

### 3.2. Changes of Pulmonary TUNEL-Positive Cells

**Figure 2:**
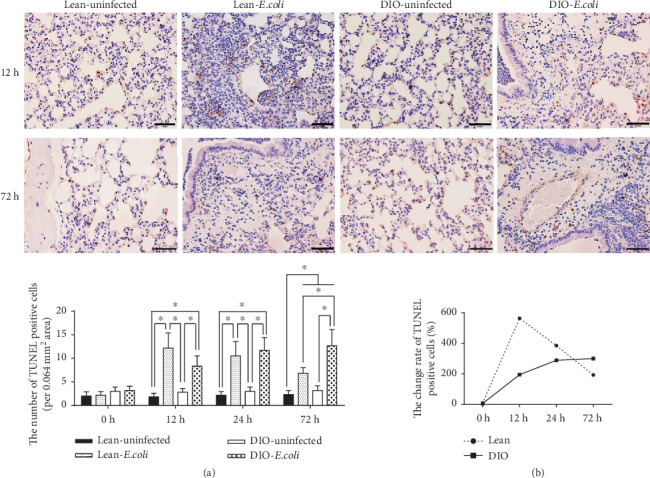
The pulmonary cell apoptosis by TUNEL. The representative images of TUNEL-positive cells of the lung following *E. coli* infection at 12 h or 72 h after infection; (a) The number of TUNEL-positive cells; (b) The change rate of TUNEL-positive cells. Scale bar = 50 *μ*m. Note: Symbol ^∗^ represents the significant difference (*p* < 0.05).

There were no significant differences of TUNEL-positive cells between the lean- and DIO-uninfected groups from 0 h to 72 h. After infection, a large number of infiltrated neutrophils and some epithelium appeared apoptosis, and the number of TUNEL-positive cells significantly increased from 12 h to 72 h (*p* < 0.05) in the two infected groups comparing with each uninfected group. However, the changes on the number of apoptotic cells were different between the lean group and the DIO group along the time. The number peaked at 12 h in the lean-*E. coli* group, while it gradually increased in the DIO-*E. coli* group from 12 h to 72 h (Figure 2).

### 3.3. Changes of Calpain 2, Caspase 12, and JNK mRNA Relative Expressions in the Lungs

**Figure 3:**
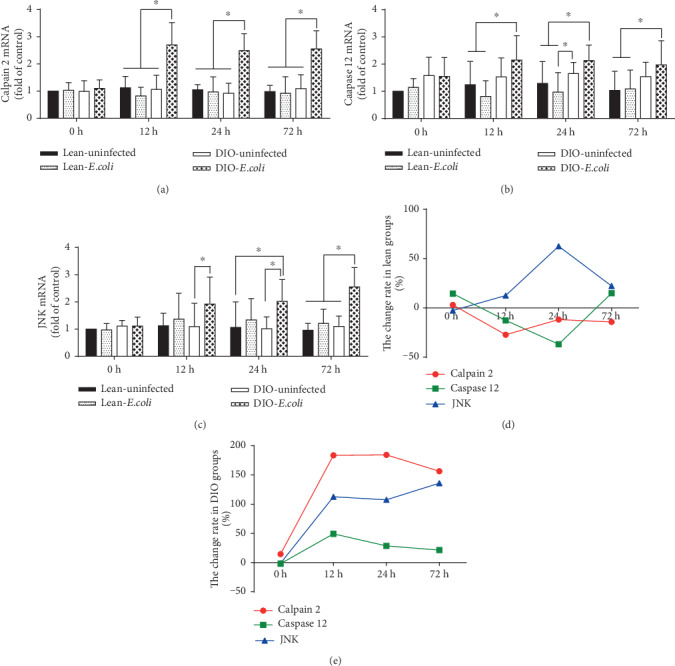
Relative mRNA expressions of endoplasmic reticulum apoptotic pathway associated apoptotic factor. (a–c) The mRNA levels of Calpain 2, Caspase 12, and JNK (fold of control); (d, e) The change rates of pulmonary apoptotic factor mRNA expression in the lean and DIO mice. Note: Symbol ^∗^ represents the significant difference (*p* < 0.05).

The relative expressions of Calpain 2, Caspase 12, and JNK mRNA exhibited no significant differences (*p* > 0.05) in the lean-*E. coli* group when compared with the lean-uninfected group at all time points. However, the relative expressions of Calpain 2 and JNK mRNA in the DIO-*E. coli* group, except Caspase 12 (*p* > 0.05), were all significantly increased (*p* < 0.05) from 12 h to 72 h after infection compared with the DIO-uninfected group (Figures 3(a)–3(c)). Moreover, the line/dot graphs (Figures 3(d) and 3(e)) indicated that only JNK increased and peaked at 24 h in the lean mice, while Calpain 2, Caspase-12, and JNK all increased from 12 h to 72 h in the DIO mice.

### 3.4. Changes of Calpain 2, *p*-Caspase 12, and *p*-JNK Relative Protein Expressions in the Lungs

**Figure 4:**
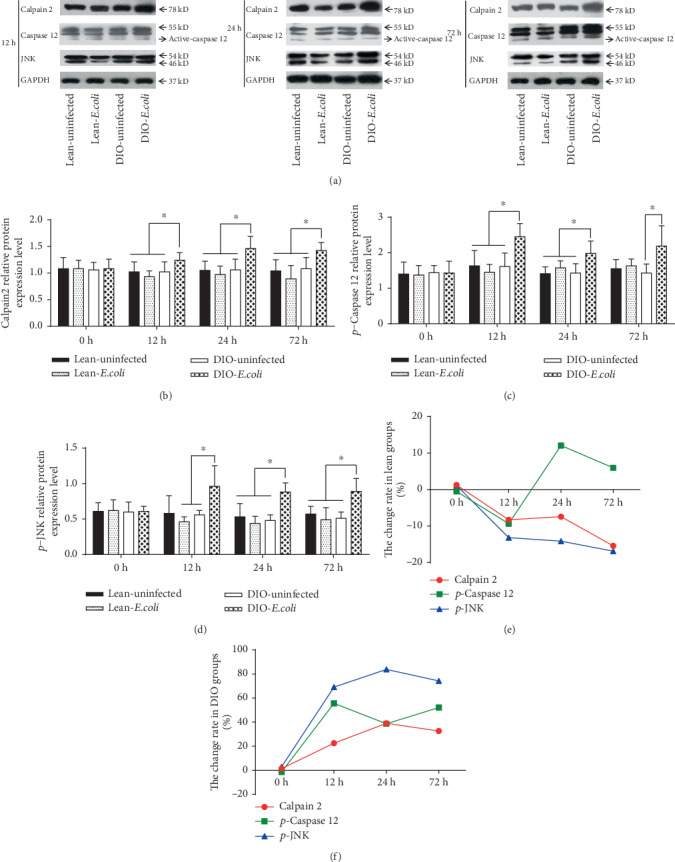
Relative protein expressions of endoplasmic reticulum apoptotic pathway associated apoptotic factor. (a) Representative western blot of protein expression; (b–d) The relative protein levels of Calpain 2, *p*-Caspase 12, and *p*-JNK (fold of control); (e, f) The change rates of pulmonary apoptotic factor relative protein expression in the lean and DIO mice. Note: Symbol ^∗^ represents the significant difference (*p* < 0.05).

Similar to mRNA, when compared with the lean-uninfected group, the relative protein expressions of Calpain 2, *p*-Caspase 12, and *p*-JNK in the lean-*E. coli* group showed no significant differences (*p* > 0.05) during the experiment. However, the relative expressions of these proteins were markedly higher in the DIO-*E. coli* group than those in the DIO-uninfected group from 12 h to 72 h (*p* < 0.05) (Figures 4(b)–4(d)). In the lean mice, only *p*-Caspase 12 increased at 24 h and 72 h (Figure 4(e)). However, in the DIO mice, Calpain 2, *p*-Caspase 12, and *p*-JNK all displayed a gradual increase from 12 h to 72 h (Figure 4(f)).

### 3.5. Changes of TNF-*α*, TNFR1, and FADD Relative mRNA Expressions in the Lungs

**Figure 5:**
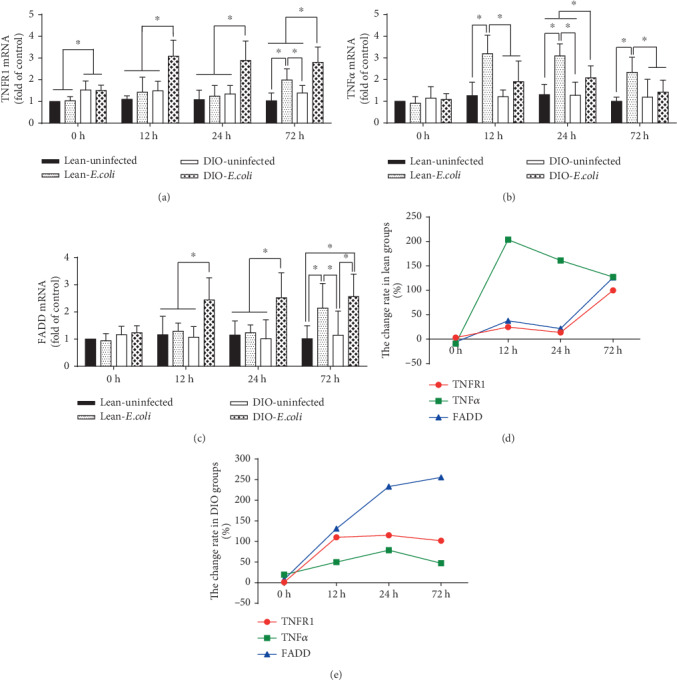
Relative mRNA expressions of apoptotic factors associated with the death receptor pathway. (a–c) The mRNA levels of TNFR1, TNF-*α*, and FADD (fold of control); (d, e) The change rates of apoptotic factor mRNA expression in the lung of lean and DIO mice. Note: Symbol ^∗^ represents the significant difference (*p* < 0.05).

Before infection, the DIO mice had a higher TNFR1 mRNA relative expression than the lean mice (*p* < 0.05). After infection, mRNA relative expressions of TNFR1 and FADD in the lean-*E. coli* group significantly increased only at 72 h in comparison with the lean-uninfected group (*p* < 0.05), while the relative expression of TNF-*α* mRNA significantly increased throughout the experiment (*p* < 0.05). By contrast, the relative expressions of TNFR1 and FADD mRNA significantly increased in the DIO-*E. coli* group from 12 h to 72 h when compared with those in the DIO-uninfected group (*p* < 0.05), and the TNF-*α* mRNA was markedly higher (*p* < 0.05) in the DIO-*E. coli* group than in the DIO-uninfected group at 24 h and 72 h (Figures 5(a)–5(c)). Moreover, from the line/dot graphs, the change rate of TNF-*α* increase from 12 h to 72 h in both the lean and DIO mice, which was more dramatic in the lean mice. The change rates of TNFR1 and FADD peaked at 72 h in the lean mice, but these change rates increased in the DIO mice from 12 h to 72 h (Figures 5(d) and 5(e)).

### 3.6. Changes of TNF-*α*, TNFR1, and FADD Relative Protein Expression in the Lung

**Figure 6:**
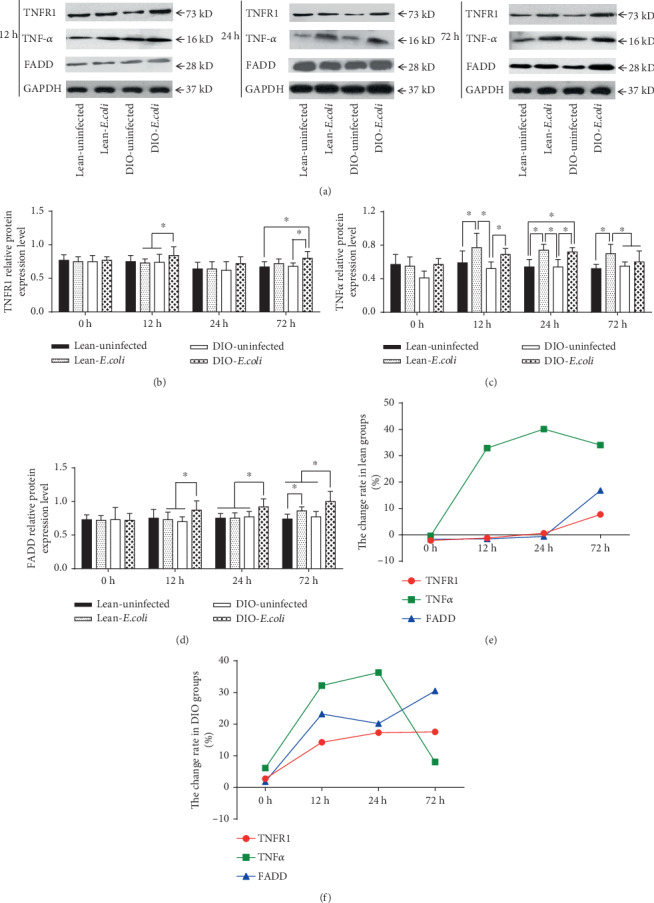
Relative protein expressions of apoptotic factors associated with the death receptor pathway. (a) Representative western blot of protein expression; (b–d) The relative protein levels of TNFR1, TNF-*α*, and FADD (fold of control); (e, f) The change rates of relative protein expression of pulmonary apoptotic factors in the lean and DIO mice. Note: Symbol ^∗^ represents the significant difference (*p* < 0.05).

Compared with the lean-uninfected group, the relative expression of TNF-*α* protein significantly increased in the lean-*E. coli* group from 12 h to 72 h, whereas the relative expression of FADD protein significantly increased only at 72 h (*p* < 0.05). However, when compared with the DIO-uninfected group, the relative expression of FADD (12 h to 72 h), TNFR1 (12 h and 72 h), and TNF-*α* (12 h and 24 h) evidently increased (*p* < 0.05) in the DIO-*E. coli* group (Figures 6(b)–6(d)). Moreover, in the lean mice, the change rate of TNF-*α* increased from 12 h to 72 h, whereas TNFR1 and FADD increased after 24 h (Figure 6(e)). In addition, the change rates of TNF-*α*, TNFR1, and FADD increased in the DIO mice from 12 h to 72 h (Figure 6(f)).

### 3.7. Subcellular Localization of Endoplasmic Reticulum and Death Receptor Apoptotic Pathway Relative Proteins in the Lungs

**Figure 7:**
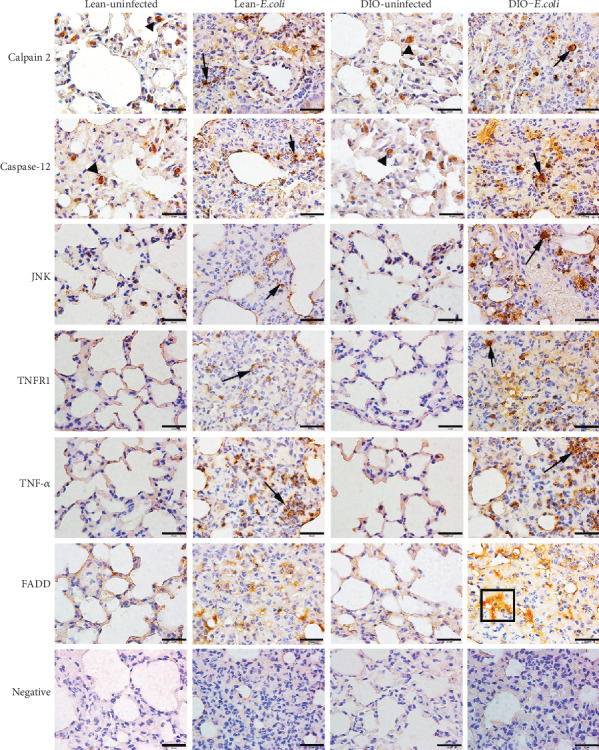
Representative immunohistochemistry staining of apoptotic proteins associated with the endoplasmic reticulum and death receptor pathway at 24 h. DAB, scale bar = 20 *μ*m. Positive Calpain 2, Caspase-12, JNK, TNFR1, and TNF-*α* proteins in neutrophils (long arrows); Positive Calpain 2 or Caspase-12 proteins in alveolar epithelia (arrowheads); Positive FADD proteins in the neutrophil infiltrated areas (boxes).

The immunohistochemistry staining was here to confirm the locations of Calpain 2, Caspase 12, JNK, TNFR1, TNF-*α*, and FADD in this research. As shown in Figure 7, in the uninfected groups, all these proteins showed slight positive staining in the cytoplasm of pulmonary epithelia, while Calpain 2 and Caspase 12 showed positive staining in the alveolar septum. After infection, large numbers of positive proteins were detected in the cytoplasm of inflammatory cells and pulmonary epithelia in the neutrophil-infiltrated areas. Moreover, the positive expressions of these proteins were higher in the DIO-*E. coli* group than those in the lean-*E. coli* group, except TNF-*α* with high expressions in both groups.

## 4. Discussion

Obesity has become a worldwide health problem in the twenty-first century. In 2010, overweight and obesity were estimated to cause 3.4 million deaths [21]. The prevalence rates of overweight are particularly high in the Americas, Europe, and Middle East, and obesity among school-age boys/girls by global region [22]. Obesity increases morbidity and mortality from many chronic health ailments, such as cardiovascular disease, type II diabetes, dyslipidemia, and fatty liver disease [23]. However, obese individuals have a paradoxical response to bacterial pneumonia. Although they are more sensitive, they have improved outcomes, like reduced mortality [24–26]. Similar to this phenomenon, in our previous study, the DIO mice exhibited a delayed inflammatory response and oxidative stress, as well as pulmonary cell apoptosis through the mitochondria-mediated pathway. For further study, ER stress- and death receptor-mediated apoptotic pathways were detected.

To verify a successful pneumonia model, histopathological observation was executed. After *E. coli* infection, as described in the results, large numbers of neutrophils and macrophages infiltrating into the alveolus illustrated acute pneumonia occurred in both lean- and DIO-*E. coli* groups.

In bacterial pneumonia, neutrophils are firstly recruited from the systemic circulation into the site of tissue injury or infection, then directly kill microbes by phagocytosis, degranulation, and production of reactive oxygen species (ROS) [27]. As well known, ROS is not only inevitable by-products of oxygen metabolism, but also plays a role in cellular signaling. Excessive ROS can induce apoptosis through both the extrinsic and intrinsic pathways [28]. Moreover, neutrophils that migrate into the inflammation areas to ingest and kill bacteria would be removed either by necrosis or by apoptosis [9, 29]. Numerous researches have reported increased apoptosis of neutrophils during pneumonia or infection [9, 29, 30]. Our previous study noticed an increased cell apoptosis in the *E. coli*-infected lung through flow cytometry [17]. In the present study, by TUNEL staining, the results showed that the pulmonary cell apoptosis increased in the lean- and DIO-*E. coli* groups during the experiment and suggested the major apoptotic cells could be neutrophils. Then, this study puts emphasis on the endoplasmic reticulum-induced apoptotic pathway and death receptor pathway between the lean and DIO mice.

ER stress response is induced by Ca^2+^ homeostasis and accumulation of unfolded proteins in the ER, and prolonged ER stress triggers the cellular UPR, which results in an increase in ER stress-induced apoptotic transcription factors [31, 32]. c-Jun N-terminal kinase (JNK), belonging to the mitogen-activated protein kinase family, is involved in apoptosis, inflammatory condition, and cytokine production (such as IL-8) [33, 34]. There are several reports regarding the mechanism of JNK-induced apoptosis in response to TNF-*α* [35]. Moreover, it has been reported that oxidative stress can activate the JNK signal transduction pathway in several cell types [36]. Calpain 2, belonging to the family of calcium-dependent intracellular cysteine proteases, is expressed ubiquitously in mammals and many other organisms [37]. ROS, produced from the ER or other sources, can target the ER calcium channels inducing calcium release [38]. Once intracellular calcium homeostasis is disordered, calpain-mediated cleavage and activation of caspase-12 would be initiated. Different proapoptotic stimuli, including TNF and lipopolysaccharide treatments, cause caspase-12 processing, then lead to the apoptotic progression [39].

As reported in our previous study, after *E. coli* infection, there were continuous increases of the pulmonary cytokine levels, especially TNF-*α* and IL-8, and oxidative stress levels in the lean-and DIO-*E. coli* groups [18]. In accordance with these results, our present research showed that the mRNA and protein expressions of *p-*JNK, Caspase 12, and Calpain 2 were upregulated gradually in the DIO-*E. coli* group from 12 h to 72 h. In contrast, although lean mice also showed significantly increased cytokines and oxidative stress, there were no significant changes on the apoptotic parameters related to ER stress. Moreover, the level of pulmonary resistin, in the acute bacterial pneumonia, was significantly decreased in the DIO mice, not in the lean mice [18]. Lefterova et al. found that ER stress may underlie the downregulation of resistin mRNA and protein in murine obesity [40]. These data suggested that the DIO mice suffered from more ER stress and ER stress-mediated apoptosis than the lean mice.

Death receptor-activated apoptotic pathway is an “extrinsic pathway”, which is activated by ligand-bound death receptors, such as TNF, Fas, or TRAIL receptors. The death receptors contain an intracellular globular protein interaction domain called the death domain (DD). Upon ligand binds to death receptors probably in the form of preassociated receptor trimers, the activated death receptors recruit an adaptor protein, called Fas Associated Death Domain (FADD) [41]. TNF-*α* mediates its effects by binding to either of two receptors, TNFR1 and TNFR2, and TNFR1 has a death domain that promotes apoptosis, whereas TNFR2 does not [42]. In rodent *E. coli* pneumonia, *E. coli* and *E. coli*-LPS stimulated TNF-a production by alveolar macrophages [43]. Thus, the present study also tested the TNF-*α*/TNFR pathway. The relative mRNA and protein expressions of TNF-*α*, TNFR1, and the adaptor protein FADD were all significantly increased in the DIO-*E. coli* group after infection. These indicated that *E. coli* pneumonia-induced cell apoptosis in the lung was partly executed via the TNF-*α*-mediated death receptor apoptotic pathway in the DIO mice. However, in the lean mice, the pulmonary TNFR1 and FADD levels significantly increased only at 72 h postinfection. As reported previously, neutrophil infiltration is the main characteristic of acute bacterial pneumonia, and neutrophils are able to express proinflammatory cytokines, including TNF-*α* [44, 45]. And in the *E. coli* pneumonia, acute inflammation response (like neutrophil recruitment and emigration) was not compromised by the gene-targeted deficiency of both TNFR1 and TNFR2 [46, 47]. Therefore, the upregulation of TNF-*α* by neutrophils might be TNFR independent in the bacterial pneumonia. Moreover, TNFR1 had an adequate response to leptin generation and obesity establishment in mice fed a high-fat diet, and knockout of TNFR1 protected genetically obese *ob/ob* mice from insulin resistance [48, 49]. As shown in the results, the DIO mice exhibited a higher level of TNFR1 mRNA than the lean mice, and greater changes on mRNA and protein expressions after infection. These all above indicated that the DIO mice might have a more sensitive response of the changes in TNFR1.

Although the pulmonary expressions of apoptotic factors associated with ER stress and death receptor in the DIO mice were higher than those in the lean mice, the DIO mice exhibited less TUNEL-positive cells. The conflicted results could be contributed by leptin, one adipokine secreted by adipocytes. It has been reported that leptin could inhibit PERK- (PKB like ER kinase-) mediated ER stress and apoptosis [50]. DIO mice possess of higher leptin level than the lean mice before and after infection, which suggested that the pulmonary apoptosis mediated by ER stress could be suppressed by leptin. Moreover, after pulmonary infection, the pathway involved in apoptosis could be different between the lean mice and DIO mice. Our early research revealed that the lean mice exhibited significant pulmonary apoptosis via mitochondrial apoptotic in the lean mice after acute bacterial pneumonia [17], other than the ER stress- or death receptor-mediated pathway activated in the DIO mice, which may partly explain the results.

For a subcellular localization of these apoptotic factors, immunohistochemistry was performed. Phosphorylation of JNK takes place in cytoplasm after it bounds to the COOH-terminal cytoplasmic portion of the transmembrane protein kinase IRE1 (inositol-requiring enzyme) [51]. As mentioned above, ROS induced by ER stress could cause calcium release from ER; then, mitochondria take up the released calcium and produce more ROS [38]. Calpain 2, a Ca^2+^-sensitive cysteine protease, is elevated with the increase of cytosolic Ca^2+^ [52]. Caspase-12 is a protein that belongs to caspase proteins, and the proenzyme of caspase-12 is activated under the stimulation of calpain in the cytosol [39, 53]. After *E. coli* infection, JNK, Calpain 2, and Caspase-12 proteins were displayed mainly in the cytoplasm of inflammatory cells and pulmonary epithelial cells in the infected areas. TNF-*α*, a cell-signaling protein, is produced chiefly by activated macrophages, and many other cell types, such as lymphocytes, neutrophils, and mast cells [54]. TNFR is the death receptor of the ligand TNF*α* [42]. Thus, after infection, TNF*α* and TNFR1 proteins were mainly displayed in the cytoplasm of inflammatory cells. FADD is a 23 kDa adaptor protein that bridges TNFR1 to procaspases 8 to form the death-inducing signaling complex (DISC) during apoptosis [55]. The staining of FADD was located in the cytoplasm or cell surface [56]. In this study, FADD positive protein was detected as dispersive distribution in the cytoplasm or cell surface of inflammatory cells or epithelial cells in the lean- and DIO-*E. coli* groups.

## 5. Conclusions

In conclusion, after infected with *E. coli*, both the lean and DIO mice exhibited increased percentages of apoptosis cells in the lung, and there were more apoptotic cells in the lean mice before 24 h postinfection, which supports the “obese paradox”. Most impressively, the pulmonary apoptosis was mainly mediated by ER stress and death receptor in the DIO mice with acute bacterial pneumonia, while it did not occur in the lean mice.

## Figures and Tables

**Table 1:** Sequence of primers used in qRT-PCR.

Target gene	Accession number	Primer sequence (5′-3′)	Product size
Calpain 2	NM_009794.3	Forward: AGATGCGGAAAGCACTGGAA Reverse: GGACCAAACACCGCACAAAA	126 bp
Caspase 12	NM_009808.4	Forward: GGGTTTTTGATGACCTGGTGG Reverse: GCCAATCCAGCATTTACCTCC	298 bp
JNK	NM_001310453.1	Forward: TCATTCTCGGCATGGGCTAC Reverse: CCTGGGAACAAAACACCACC	94 bp
TNF-*α*	NM_013693.3	Forward: ACTGGCAGAAGAGGCACTCC Reverse: CTGCCACAAGCAGGAATGAG	95 bp
TNFR1	NM_011609.4	Forward: CCTGACAATGCAGACCTTGC Reverse: CTCCAGCCTCTCGATCTCGT	117 bp
FADD	NM_010175	Forward: GATGGATGGGATTGAGGAGA Reverse: CCAGGTCAGCCACCAGATT	155 bp
*β*-Actin	NM_007393	Forward: GCTGTGCTATGTTGCTCTAG Reverse: CGCTCGTTGCCAATAGTG	117 bp

## Data Availability

The cytokines contents and oxidative stress data used to support the findings of this study have been deposited in the PubMed repository (10.1038/s41598-018-32420-3). The mitochondrial apoptotic pathway data and flow cytometry data used to support the findings of this study have been deposited in the PubMed repository (10.1155/2019/1968539). The qRT-PCR and Western bolt data used to support the findings of this study are included within the article.
